# Invasive versus Non-invasive Positive Pressure Ventilation In Chronic Obstructive Pulmonary Disease Complicated By Acute Respiratory Failure

**DOI:** 10.7759/cureus.5418

**Published:** 2019-08-18

**Authors:** Pooja Devi, Ravi Raja, Ram Kumar, Ali Shah, Sanober I Ansari, Besham Kumar

**Affiliations:** 1 Internal Medicine, Ziauddin University, Karachi, PAK; 2 Internal Medicine, New Medical Center, Al Ain, ARE; 3 Internal Medicine, Chandka Medical College, Larkana, PAK; 4 Internal Medicine, Jinnah Sindh Medical University, Karachi, PAK; 5 Family Medicine, Emirates Airline Medical Services, Dubai, ARE; 6 Internal Medicine, Jinnah Postgraduate Medical Center, Karachi, PAK

**Keywords:** chronic obstructive pulmonary disease, acute exacerbation of copd, acute respiratory failure, noninvasive ventilation, mechanical ventilation, invasive ventilation, mortality, survival, blood gas analysis

## Abstract

Introduction

Acute exacerbation of chronic obstructive pulmonary disease (AECOPD) is frequently encountered as a medical emergency. AECOPD is the third leading medical cause of hospitalization due to acute respiratory failure (ARF). The utilization of ventilators for patients with ARF secondary to AECOPD has increased. There has been a major inclination towards utilization of non-invasive positive pressure ventilation (NIPPV) and sparing invasive positive pressure ventilation (IPPV) for life-threatening respiratory distress and/or in patients where NIPPV failure is observed. The aim of this observational study was to compare the clinical and laboratory parameters patients with chronic obstructive pulmonary disease (COPD) complicated by ARF admitted in the intensive care unit (ICU).

Methods

In the prospective observational study with known cases of COPD complicated by ARF, patients were grouped into NIPPV and IPPV groups based on their clinical and laboratory parameters. Thirty patients were included in each group. Demographic data was collected. Clinical and laboratory parameters were evaluated at baseline and at 24 hours of ventilation. The outcome was assessed in terms of duration of ventilation, hospital and ICU stay and overall mortality. Data was entered and analyzed using SPSS version 22.0 (IBM Corp., Armonk, NY).

Results

Both IPPV and NIPPV groups demonstrated marked reduction in partial pressure of carbon dioxide (PaCO_2_) with 24 hours of ventilation (for IPPV: 78.1 ± 20.2 vs. 69.1 ± 20.2; p=0.08) (for NIPPV: 68.1 ± 17.8 vs. 57.2 ± 21.5; p=0.03). In NIPPV group, there was significant improvement in partial pressure of oxygen (PaO_2_) (p=0.009), respiratory rate (p=0.008), heart rate (p<0.0001), systolic blood pressure (p=0.03), and diastolic blood pressure (p<0.0001). These parameters did not improve significantly in the IPPV group except for systolic blood pressure (p=0.008). The NIPPV failure rate was 20%. NIPPV patients had a significantly shorter duration of ventilation, ICU stay, and hospital stay. In-ICU mortality was significantly lower in the NIPPV group as compared to IPPV (13% vs. 40%; p=0.01). There was no difference in post-ICU in-hospital mortality between the two groups (6.7% vs. 16.7%; p=0.13).

Conclusion

Both NIPPV and IPPV are effective in normalizing acidosis and hypercapnia in patients with COPD complicated by ARF. Patients managed with non-invasive mode of ventilation have a shorter duration of ICU as well as hospital stay. Survival rates are also better as compared to patients managed with invasive ventilation.

## Introduction

Chronic obstructive pulmonary disease (COPD) is a major public health concern. Globally, 328 million people are suffering from COPD. COPD is the third leading cause of global mortalities and 90% of COPD related deaths occur in low-to-middle-income countries [1]. Acute exacerbation of COPD (AECOPD) is frequently encountered as a medical emergency. It is associated with reversible alterations in pulmonary mechanics leading to impaired gaseous exchange and, hence, severe respiratory distress. AECOPD is the third leading medical cause of hospitalization due to acute respiratory failure (ARF) [2]. Of all the hospitalizations with ARF due to AECOPD, 12%-18% patients need admission in the intensive care unit (ICU) [3]. The overall rate of mortality in AECOPD is approximately 30% [4]. The mortality rate in COPD patients with ARF approaches 10%-15% [4-5].

Over the years, the utilization of ventilators for patients with ARF secondary to AECOPD has increased. There has been a major inclination towards utilization of non-invasive positive pressure ventilation (NIPPV) and sparing invasive positive pressure ventilation (IPPV) for life-threatening respiratory distress and/or in patients where NIPPV failure is observed [2]. Systematic reviews of randomized controlled trials have established that NIPPV, in treatment of ARF secondary to AECOPD, reduces the need for intubation, rate of treatment failure, duration of ICU stay as well as hospital stay, and overall mortality [6-9]. NIPPV also demonstrated rapid improvement in pH, partial pressure of carbon dioxide (PaCO_2_), and respiratory rate [7].

Although NIPPV has reduced rate of complications, this mode of ventilation can only be administered in patients who are conscious, able to cooperate (not irritated and/or agitated), and not in severe respiratory distress. In patients with severe ARF, altered consciousness, unstable vital signs, severe cardiac arrhythmia, or requiring emergency intubation; IPPV is indicated [10]. Although, complications associated with mechanical ventilation and intubation-ventilator associated pneumonia, pulmonary edema, pleural effusion, and atelectasis - cannot be neglected [11]. There is published literature in relation to the efficacy of NIPPV in comparison to standard medical care and also in comparison to IPPV; the impact of NIPPV and IPPV on clinical and biochemical profile of patients with acute respiratory failure secondary to COPD is not readily documented. For this purpose, a prospective observational study was conducted to compare the clinical and laboratory parameters of respiratory failure in patients with respiratory failure admitted in ICU.

## Materials and methods

It was randozmied, prospective, observational study conducted in the ICU of the pulmonology unit of Civil Hospital, Jamshoro, Pakistan. Known cases of COPD admitted in the ICU with acute respiratory failure from January - December 2018 were recruited in the study. Patients with both hypercapnic (PaCO2 > 50 mmHg; pH < 7.30) and hypoxemic (PaO2 <60 mmHg) respiratory failure [12] were included. Choice of NIPPC or IPPV was made by the treating pulmonologist and critical care specialists based on the clinical findings and biochemical derangements. Selection and exclusion criteria for Non-Invasive Positive Pressure Ventilation (NIPPV) and indications of Invasive Positive Pressure Ventilation (IPPV) are summarized in Table 1 adapted from Pauwels et al. [13].

**Table 1:** Selection and exclusion criteria for NIPPV and indications of IPPV IPPV, Invasive Positive Pressure Ventilation; NIPPV, Non-invasive Positive Pressure Ventilation; PaO2, Partial Pressure of Oxygen; PaCO2, Partial Pressure of Carbon dioxide.

Criteria	Severity
Selection criteria for NIPPV (at least two should be present)	Moderate to severe dyspnea with use of accessory muscles and paradoxical abdominal motion
	Moderate to severe acidosis (pH 7.30–7.35) and hypercapnia (PaCO_2_ 45–60 mmHg)
	Respiratory frequency > 25 breaths/minute
	Moderate to severe hypoxemia (PaO_2_ < 60 and PaCO_2_ <45 mmHg)
Exclusion criteria for NIPPV (any of these may be present)	Respiratory arrest
	Cardiovascular instability (hypotension, arrhythmias, myocardial infarction)
	Somnolence, impaired mental status, uncooperative patient
	High aspiration risk; viscous or copious secretions
	Extreme obesity
	Recent facial or gastroesophageal surgery
	Craniofacial trauma, fixed nasopharyngeal abnormalities
Indications of IPPV	Severe dyspnea with use of accessory muscles and paradoxical abdominal motion
	Respiratory frequency > 35 breaths/min
	Life-threatening hypoxemia (PaO_2_ 40 mmHg)
	Severe acidosis (pH < 7.25) and hypercapnia (PaCO_2_ > 60 mmHg)
	Respiratory arrest
	Somnolence, impaired mental status
	Cardiovascular complications (hypotension, shock, heart failure)
	Other complications (metabolic abnormalities, sepsis, pneumonia, pulmonary embolism, barotrauma, massive pleural effusion)
	NIPPV failure

Demographic data - age and gender - was included for all participants. Clinical data collected included respiratory rate, heart rate, blood pressure, pH, PaCO2, and PaO2. All parameters were assessed at baseline and then repeated at 24 hours of initiation of treatment. The outcome was evaluated in terms of duration of ventilation, length of ICU stay, length of hospital stay, mortality within the ICU or post-ICU within the hospital, and failure of NIPPV requiring a shift to IPPV. For statistical analysis, SPSS for Windows version 22.0 (IBM Corp., Armonk, NY). Categorical variables were presented as frequencies and percentages. Continuous variables were presented as mean and standard deviation (SD). Chi-square test was used for categorical variables and independent student t-test for quantitative variables. P value of ≤ 0.05 was taken as significant.

## Results

In each group, 30 patients completed the study. Their mean age was 67.4 ± 21.8 years. Patients in the NIPPV group were younger than patients in the IPPV group (p=0.01). There were 39 (65%) men and 21 (35%) women.

PaCO2, PaO2, respiratory rate, heart rate, and blood pressure of NIPPV and IPPV groups at baseline (before any medical intervention) and after 24 hours of ventilation are shown and compared in Table 1. Table 1 shows that in the NIPPV group, PaCO2 reduction with 24 hours of ventilation was not statistically significant (p=0.28); in the IPPV group, this reduction was significant (p=0.08). In the NIPPV group, PaO2 improvement with 24 hours of ventilation was statistically significant (p=0.009); in the IPPV group, this improvement was not significant (p=0.23). Improvement in respiratory rate was statistically significant (p=0.008) in the NIPPV group, however, not significant (p=0.21) in IPPV group. Improvement in heart rate was only statistically significant (p<0.0001) in the NIPPV group. Statistically significant reduction in systolic blood pressure was seen in both groups; diastolic blood pressure reduction was only seen in the NIPPV group. All variables of the demographic and clinical profile are shown in Table 2.

**Table 2:** Comparison of demographic and clinical characteristics of patients on invasive and non-invasive positive pressure ventilation * For both groups (NIPPV and IPPV), values at baseline were compared with those after 24 hours of ventilation BP, Blood Pressure; IPPV, Invasive Positive Pressure Ventilation; NIPPV, Non-invasive Positive Pressure Ventilation; PaO2, Partial Pressure of Oxygen; PaCO2, Partial Pressure of Carbon dioxide; SD, Standard Deviation.

Patients Characteristics	At baseline			At 24 hours of ventilation			P value within groups*	
	NIPPV (n=30)	IPPV (n=30)	P value	NIPPV (n=30)	IPPV (n=30)	P value	NIPPV (n=30)	IPPV (n=30)
Gender				----------				
Male n (%)	18 (60%)	21 (70%)	0.41					
Female n (%)	12 (40%)	9 (30%)						
Age, years	65.4 ± 11.8	73.6 ± 13.2	0.01					
PaCO_2_, mmHg	63.2 ± 21.5	78.1 ± 20.2	0.007	57.2 ± 21.5	69.1 ± 20.2	0.03	0.28	0.08
PaO_2_, mmHg	61.3 ± 18.9	60.2 ± 21.4	0.83	74.6 ± 19.3	65.7 ± 13.4	0.04	0.009	0.23
Respiratory rate per minute	27.4 ± 3.1	29.7 ± 4.8	0.03	25.2 ± 3.1	27.9 ± 6.2	0.03	0.008	0.21
Heart rate per minute	113.4 ± 9.7	121.1 ± 11.3	0.006	103.2 ± 6.1	118.4 ± 9.5	<0.0001	<0.0001	0.32
Systolic BP, mmHg	146.2 ± 17.3	137.9 ± 13.4	0.04	138.1 ± 10.7	130.2 ± 7.5	0.001	0.03	0.008
Diastolic BP, mmHg	68.5 ± 15.6	86.1 ± 18.1	0.0002	84.2 ± 11.8	92.4 ± 15.3	0.02	<0.0001	0.15

Patient outcome was compared for both groups. NIPPV group showed a significantly shorter duration of ventilation days, IUC days, and post-ICU hospitalization days. In-ICU mortality rate for NIPPV group was 13.3% and for IPPV group it was 40% (p=0.01). Post-ICU mortality for NIPPV group was 6.7% and for IPPV group it was 16.7%; however, the differences were not statistically significant. No mortality was reported within the first 24 hours of hospitalization. In 20% (n=6) of NIPPV patients, non-invasive ventilation failed; they were transferred to invasive ventilation. Of these six patients, 3 (50%) died within the ICU; rest recovered and were discharged. There was no case of readmission to the ICU or need for re-ventilation after extubation. All parameters of patient outcome between the two groups are shown in Table 3.

**Table 3:** Comparison of outcome of patients on invasive and non-invasive positive pressure ventilation ICU, Intensive Care Unit; IPPV, Invasive Positive Pressure Ventilation; NIPPV, Non-invasive Positive Pressure Ventilation; SD, Standard Deviation.

Patient Outcome	NIPPV (n=30) (Mean ± SD)	IPPV (n=30) (Mean ± SD)	P value
Duration of ventilation, days	4.9 ± 1.8	6.1 ± 2.5	0.03
Duration of ICU stay, days	10.3 ± 1.3	17.3 ± 2.1	<0.0001
Post-ICU hospital stay, days	13.4 ± 8.7	22.9 ± 6.7	<0.0001
In-ICU mortality n (%)	4 (13.3%)	12 (40%)	0.01
Post-ICU mortality n (%)	2 (6.7%)	5 (16.7%)	0.13
Failure of NIPPV n (%)	6 (20%)		

## Discussion

Clinical parameters of IPPV and NIPPV groups was comparable. IPPV group showed better improvement in PaCO2 and NIPPV group better improvement in PaO2 over 24 hours. IPPV group did not show a statistically significant reduction in respiratory and heart rate. Both groups showed improvement in systolic BP and only NIPPV showed improvement in diastolic BP. On all parameters - days of ventilation, days of ICU stay, days of post-ICU stay, and in-ICU mortality - patient outcome was significantly better in the NIPPV group.

To the best of our knowledge, this study remains the first comparison between invasive and non-invasive ventilation in ARF secondary to COPD exacerbation from Pakistan. Previously, the outcome of non-invasive ventilation has been reported from this region [14-15]; however, the comparison is reported for the first time in this study. Primary diagnosis was prospectively assigned at the time of admission by trained critical care specialists and pulmonologists; instead of retrospectively by coders. All laboratory parameters were evaluated prospectively. The study has its limitations too. The most prominent limitation is its sample size. Since the study was based in one center only, not many patients could be recruited during the study period. Its small sample size has a limited establishment of any concrete relationships. For assessing the outcome, no standard score such as "Acute Physiology, Age, Chronic Health Evaluation II" (APACHE II) Score was utilized. Complications associated with NIPPV and IPPV were not evaluated, hence, the safety of either mode of ventilation cannot be established through this study.

In 2016, Maleh et al. compared the therapeutic efficacy and outcome of COPD patients admitted with ARF in the ICU [10]. With 24 hours of medical intervention, percent improvement in respiratory rate, heart rate, pH, PaCO2, PaO2 in the NIPPV group was lower than in the IPPV group. The average length of hospital stay in IPPV was longer than NIPPV {15.90 ± 10 vs. 8.12 ± 6.49 days (p<0.05)}. Mortality rate in the NIPPV was lower than IPPV {8% vs. 54% (p <0.05)}. NIPPV group better outcome in terms of hospital stay and mortality, however, it cannot be neglected that IPPV patients were more severely ill [10]. Conversely, in this study, the improvement in clinical and laboratory parameters of both groups were comparable after 24 hours. NIPPV group in this study also showed reduced ICU and hospital stay and better survival.

NIPPV is effective and can be utilized as an alternative to intubation in patients with ARF. Venkatram et al. compared NIPPV and IPPV in patients with AECOPD admitted to ICU [16]. NPPV ventilation was administered to 41% of patients with ARF secondary to AECOPD. It was successful in 94.5% patients and failure of NIPPV was observed in 5.5%. There was no mortality in this group. In comparison, out of 59% patients with IPPV, the mortality rate was 3%. Patients in the NIPPV group also had a significantly shorter duration of hospital stay [16]. We reported a higher percentage of NIPPV failure (20%) and a higher mortality in both IPPV (56% vs. 3%) and NIPPV (20% vs. 0%) groups as compared to Venkatram et al. [16]. As compared to Maleh et al., our mortality rate in IPPV (56% vs. 54%) and NIPPV group (20% vs. 8%) was also higher. Rate of NIPPV failure was also higher in our study (20% vs. 10%) [10]. On the other hand, in another study from Pakistan conducted from 2001-2005, the mortality rate in NIPPV was 23.5% [15]. Statistically significant improvements were observed in the pH and PaCO2 at 24 hours and 48 hours compared to baseline. The rate of NIPPV failure was 12.6% [15].

In Lindenauer et al., although the patients in NIPPV group were older, had a lower risk of pneumonia, shorter length of stay, less cost, and less risk of mortality; their 30-day readmission rate was similar to that of IPPV group. In this study, patients younger than 85 years and those with early treatment initiation were more likely to benefit from NIPPV and patients with higher co-morbidity and concomitant pneumonia were less likely to benefit from NIPPV [17]. In another randomized prospective study, NIPPV was compared with IPPV in COPD patients admitted with ARF which stated that intubation was required in 52% patients randomized to NPPV. In the NPPV group, those who avoided intubation had a shorter duration of mechanical ventilation and ICU stay. Those who needed intubation had a comparable duration of mechanical ventilation and ICU stay as the IPPV group [18].

Even in ARF due to conditions other than COPD, NIPPV has shown marked improvement in patient outcome as compared to IPPV. In a meta-analysis of 13 observational studies with immuno-compromised patients admitted in the ICU with ARF, NIPPV showed significantly reduced in-hospital mortality (odds ratio (OR): 0.43, p value =0.007) and 30-day mortality (OR 0.34, p value <0.0001) [19]. In another meta-analysis with more than 2000 patients with do-not-intubate (DNI) and comfort-measures-only orders, non-invasive ventilation was given. The pooled survival rate was 56% at discharge and 32% at one year for DNI patients. In COPD patients with DNI orders, hospital survival rate was 68% with NIPPV [20].

This study has significant results. The benefits of non-invasive ventilation are clear in terms of providing essential outcomes such as vital stability and survival. Non-invasive ventilation should be a routine critical care intervention in patients with ARF due to COPD. However larger scale, multi-center studies are required to further investigate and consolidate these findings.

## Conclusions

Significant evidence has been attained to establish the therapeutic efficacy of non-invasive ventilation in patients with acute exacerbations of COPD complicated by respiratory failure. NIPPV is effective in normalizing acidosis and hypercapnia in these patients. Patients managed with the non-invasive mode of ventilation have a shorter duration of ICU as well as hospital stay. Survival rates are also better as compared to patients managed with invasive ventilation. However, in cases of severe ARF and hemodynamic instability, intubation and mechanical ventilation may be inevitable.
